# Book Reviews

**Published:** 1894-03

**Authors:** 


### Book Reviews.

A System of Genito-Urinary Diseases, Syphilology and Dermatology. By various authors. Edited by Prince A. Morrow, A. M., M. D., Clinical Professor of Genito-Urinary Diseases; formerly Lecturer on Dermatology in the University of the City of New York; Surgeon to the Charity Hospital, etc. With illustrations. In three large 8vo volumes. Volume II., Syphilology. Pp. xviii.—917. New York: D. Appleton & Co. 1893. Sold only by subscription.

The first volume of this system was noticed somewhat in extenso in the June number of the Journal. We now proceed to give our readers the benefit of an examination of the second volume. The editor states that in its preparation it has been the aim to produce a complete and systematic treatise on syphilis and chancroid, which should reach up to the present and embody the most recent advances made in our knowledge of these diseases. We think he has accomplished his object, while at the same time he has given to the profession a work that is essentially practical. In order to reach this end it has been necessary to eliminate many theoretical questions and unimportant details, that only the essential facts of present knowledge might be presented, and that only questions of live, practical issue might be discussed.

The history, geographical distribution, evolution and general pathological anatomy of syphilis, by James Nevins Hyde, forms the opening section of the book, and the thirty-eight pages devoted to it are of exceeding interest. The next section treats of the etiology of syphilis, and is written by John A. Fordyce. The micro-örganisms in syphilis here have received careful consideration, which is the product of elaborate research and studies in laboratory cultures. This author very properly classes, among predisposing causes, improper, or imperfectly carried out, treatment during the early stage of the disease as the most frequent causes of the recurrence of tertiary symptoms. This statement ought to receive great emphasis, for we believe that in the present state of our knowledge syphilis ought never to pass beyond the primary stage, and an enlightenment of the community on this subject should be made the aim and duty of every physician.

The modes of infection in syphilis are briefly considered by L. Duncan Bulkley. He asserts that there are four methods by which syphilis can be acquired: 1. Direct contact. 2. Mediate infection. 3. Hereditary transmission. 4. Maternal infection. Bulkley does not believe that the normal secretions—milk, saliva, etc.—can communicate the disease except when they act as carriers of a pathological secretion from a syphilitic lesion.

Primary syphilis is the subject of the next section, to the consideration of which Edward Bennet Bronson devotes thirty-five pages. Here the various lesions are described and rules for diagnosis are carefully laid down. A table differentiating primary syphilis from simple chancre is especially deserving of study. Constitutional syphilis is described by Joseph Zeisler, and then we come to syphilis of the skin, which is exhaustively treated of and illustrated by the editor-in-chief, Prince A. Morrow. Probably no department of medicine has improved more of late than the study of syphilides, and the eighty-two pages here devoted to their consideration invites most careful examination and criticism. Nothing but praise can be given to Dr. Morrow's exposition of the subject. Syphilis of the appendages of the skin is dealt with by Samuel Alexander, and next comes syphilis of the mucous membranes of the mouth and tongue, by Charles W. Allen, in the course of which seven colored illustrations afford elucidation of the text.

In the next few pages, Frank Hartley treats of syphilis of the joints, bursæ tendons and aponeuroses, and then comes syphilitic affections of the bones, which are ably handled by W. R. Townsend, who devotes fifty pages to their consideration, in the course of which occur numerous illustrations. One of the technical points here is the differentiation of syphilitic and tubercular osteitis. Dr. Townsend gives a valuable table in parallel columns that serves to aid in this differentiation. Next comes the consideration of syphilis of the upper air-passages, to which John Noland Mackenzie devotes forty-eight pages, which affords most interesting reading. Visceral syphilis is ably handled by W. T. Councilman, and much light is thrown upon this important part of the subject in the fifty-two pages here devoted to it. The next section considers syphilitic affections of the rectum and anus, and James P. Tuttle has here written most clearly and comprehensively on a difficult part of the subject.

One of the most important sections—syphilis of the genito-urinary system, male and female, written by Eugene Fuller—consists of thirty-eight pages of highly interesting material. The situation of the initial lesion is usually the penis in the male and the vagina in the female, and the exact site of the infection in either case depends upon the location of the abrasion, so that it is to be asserted with confidence if a man on exposure does not have, or fails to produce, an abrasion, he escapes syphilitic infection. There is much to be unlearned with reference to the preconceived notions regarding the method of infection as well as new views to become familiar with.

Syphilis of the nervous system, by B. Jachs, and hereditary syphilis of the nervous system, by William N. Bullard, are two interesting sections that throw much light on a department of neurology that is beginning to receive deserved attention. Syphilis of the eye, by Charles Steadman Bull, who also writes upon diseases of the eye due to inherited syphilis, is an interesting field of research and is instructively handled by the author. Hereditary syphilis, by F. R. Sturgis, finds an able exponent of the subject than whom there is no one more competent to write. Dr. Sturgis is a student, an author and a clinician who enters fully into the spirit of his work whatever it may be, and in the present instance has written a most instructive monograph.

The section on diagnosis and prognosis of syphilis, by Herman G. Klotz, and the treatment of syphilis, by J. William White, deserve to be carefully studied by every physician. We have not space to analyze these sections, but regard them as deserving the closest scrutiny. Syphilis in relation to public health, by Samuel Treat Armstrong should be read by every sanitarian and public health officer. In this section are discussed the relation of prostitution to syphilis and the methods of regulation of prostitution, as well as other measures to limit the spread of syphilis.

We now come to the final and perhaps one of the most interesting, if not important, sections of the book, in which chancroid is treated of by Edward Martin in a most masterly manner, and chancroid of the anus and rectum is dealt with at the hands of James P. Tuttle. Martin's definition of chancroid is so simple that when once learned it cannot be forgotten. He says: "It is a contagious venereal ulcer, which, when uncomplicated, is not followed by constitutional symptoms." There is no longer any dispute as to the fact that chancroid cannot produce constitutional syphilis. The pyogenic microbes of chancroid cannot be inoculated upon an unbroken skin surface.

We regard this work, which is encyclopedic in character, as the ablest exposition of syphilis extant. The authors have been selected with special reference to their knowledge and experience in the several branches of the subject upon which they have written, and, conjointly with the editor, have produced the most scholarly, exhaustive and satisfactory work on the subject in the English language. We devote considerable space to its consideration on account of the importance of the subject.

---

A Treatise on the Science and Practice of Midwifery. By W. S. Playfair, M. D., F. R. C. P., Professor of Obstetric Medicine in King's College, London; Examiner in Midwifery to the Universities of Cambridge and London, and to the Royal College of Physicians. Sixth American, from the eighth English, edition. Edited, with additions, by Robert P. Harris, M. D. In one octavo volume of 697 pages, with 217 engravings and five plates. Cloth, $4.00; leather, $5.00. Philadelphia: Lea Brothers & Co. 1893.

Since 1876, Playfair has been accepted as authority in the department of obstetrics. When his first edition was issued, it was found to be such a clear exposition of the subject that Playfair's treatise was readily adopted by our colleges as a text-book. Students, therefore, became familiar with it at once, and obstetricians have followed it through its several editions with interest and satisfaction. At the time of which we speak, and during all the years previous, it is an admitted fact that students generally left the schools in greater ignorance of obstetrics than of any other branch of medicine. This is not true at present, and it is the special pride of American medicine that its graduates rarely leave college—we mean such colleges as are worth attending—without an experience of from two to six obstetrical cases. It cannot be denied that this is a totally inadequate equipment with which to enter the practice of medicine and at once assume the grave responsibilities of the lying-in chamber; but it is such a vast improvement over the old way that we cannot but feel satisfaction in the progress made and that is making.

We have noticed the several editions of Playfair as they have appeared, and are always glad to publish in our review columns the results of our examination of such good books as this. It is four years since the last American edition was issued, and during that interval much of importance has happened in the medical world in general and in the obstetrical world in particular. To keep pace with these advances, Playfair has nearly, or quite, rewritten some of the chapters in the present edition, notably those on extra-uterine pregnancy, the Cesarean section, symphyseotomy and puerperal septicemia. It is these chapters that will be most likely to challenge criticism, for in all new studies in pathology or treatment where preconceived ideas are overturned, divers opinions obtain, and only after time has elapsed can solid ground be reached.

Playfair leans to Tait's classification of extra-uterine pregnancy, who, as is well known, considers all ectopic pregnancies to be primarily tubal, and that the other varieties are developed after rupture. Bland Sutton, as is also well known, accepts Tait's position and maintains that all forms of extra-uterine gestation pass their primary stage in the Fallopian tube. Playfair cautiously—we might almost say overcautiously—says that this opinion, although it is receiving an increasing number of supporters, can not, as yet, be admitted as conclusively proved. He, therefore, thinks it best to retain, provisionally at least, the ordinary classification.

In regard to treatment, Playfair is again conservative, affirming that if diagnosis were quite certain, removal of tube and contents by abdominal section would be quite justifiable. He then goes on to enumerate the expedients that have been offered in substitution for abdominal section, or to avoid what some are pleased to regard as so formidable an operation. We think it would be better in a text-book of this kind, and in view of the present state of our knowledge, to take a decided stand in favor of operation wherever the symptoms are urgent and the diagnosis *probable.*

Symphyseotomy is a subject that is now attracting attention in obstetric circles, and what Playfair has to say regarding it will command audience. He affirms that if a woman is operated upon in *good season* and by the sub-osseous section, she should run but moderate risk of her life and her child likewise, but that, like Cesarean section, much will depend upon the length of labor and the condition of the patient when operated on for securing a successful issue. Symphyseotomy ought to be less dangerous than the Cesarean section, and nothing short of this should satisfy those who propose to substitute it for craniotomic infanticide. He affirms, moreover, that it is a less formidable operation and women make less objection to it than they do to the abdominal operation. It requires less skill in its execution, but take the whole delivery, in many cases, and it will be found that no little skill is required to secure a favorable result. We believe that the obstetrician of the future, armed with the modified Cesarean section and symphyseotomy, ought to wipe out craniotomy and make it a lost art.

In dealing with puerperal septicemia, Playfair, like all obstetric authorities, is somewhat confusing in regard to the theories of its true nature. It seems to us that the time has arrived to teach students that this is essentially a surgical disease ; that it is a true puerperal sepsis, caused by the absorption of poison through lesions of the genital tract. Furthermore, we believe that they should be taught to conduct a labor with the same precautions that would be insisted upon in a formidable surgical operation like cranial, thoracic, or abdominal section. If this were universally done, it would reduce the number of cases of so-called puerperal fever to an exiguity heretofore unheard of, and the prevention and cure of puerperal sepsis would be an accomplished fact, or as practically so as any one in medicine.

The editor of this edition has substituted the term celiotomy and the prefix celio—for the term laparatomy and the prefix laparo—as applied to abdominal surgery. We should be very glad to resort to any reasonable means to get rid of the term laparatomy and its compounds, which we regard as a mongrel in derivation and meaning ; but we cannot accept the term celiotomy as preferable to the other. It is not technically correct, because celia means something besides belly and is already used to denote other cavities of the body. Until some word can be devised which shall mean the same, let us use, as far as practicable, the term abdominal section.

This work of Playfair must ever occupy a foremost place in obstetric medicine as a safe guide to both student and obstetrician. It holds a place among the ablest English-speaking authorities on the obstetric art.

---

INTERNATIONAL CLINICS. A Quarterly of Clinical Lectures on Medicine, Neurology, Pediatrics, Surgery, Genito-Urinary Surgery, Gynecology, Ophthalmology, Laryngology, Otology and Dermatology. By professors and lecturers in the leading medical colleges of the United States, Great Britain and Canada. Edited by JOHN M. KEATING, M. D., LL.D., Colorado Springs, Col.; Fellow of College of Physicians, Philadelphia; formerly Consulting Physician for Diseases of Women to St. Agnes' Hospital; Gynecologist to St. Joseph's Hospital; Visiting Obstetrician to the Philadelphia Hospital, and Lecturer on Diseases of Women and Children, Philadelphia; Editor Cyclopedia of the Diseases of Children. Judson Daland, M. D., Philadelphia, Instructor in Clinical Medicine, and Lecturer on Physical Diagnosis and Symptomatology, in the University of Pennsylvania; Assistant Physician to the University Hospital; Physician to the Philadelphia Hospital and to the Rush Hospital for Consumption. J. Mitchell Bruce, M. D., F. R. C. P., London, England, Physician and Lecturer on Therapeutics at the Charing Cross Hospital. David W. Finlay, M. D., F. R. C. P., Aberdeen, Scotland, Professor of Practice of Medicine in the University of Aberdeen; Physician to, and Lecturer on, Clinical Medicine in the Aberdeen Royal Infirmary; Consulting Physician to the Royal Hospital for Diseases of the Chest, London. Volume III. Third series. Royal octavo, pp. xii.—356. Philadelphia: J. B. Lippincott Co. 1893.

This volume of the third series of International Clinics follows the same general plan as its predecessors with reference to variety of subjects and careful selection of the authors. Dr. George W. Gay, of Boston, speaks most interestingly and instructively on the management of patients during critical operations; Dr. John B. Hamilton, of Chicago, has an interesting lecture in this number on tuberculosis of the sacro-iliac joint and several other surgical questions, including popliteal aneurism. Dr. Alexander Haig, of London, speaks interestingly in regard to the pathology and treatment of asthma. This teacher is fast becoming recognized as an authority on internal medicine. Gastric ulcer is another subject of great importance, and Dr. James Tyson handles it with intelligence in the lecture that he devotes to its consideration. It is important for those who have obtained the previous numbers of these clinics to secure this one promptly, that the series may be completed in their libraries as fast as issued.

THE PHARMACOPEIA OF THE UNITED STATES OF AMERICA. Seventh decennial revision (1890). By authority of the National Convention for Revising the Pharmacopeia, held at Washington, A. D. 1890. Official for January 1, 1894. Published by the Committee on Revision. Octavo, pp. l.—602. Philadelphia: J. B. Lippincott Company, Printers and Binders. P. Blakiston, Son & Co., Agents. 1893.

The enormous labor required to revise the Pharmacopeia of the United States makes it impracticable to do so oftener than once in ten years. The seventh revision, made by the convention of 1890, is before us and we feel sure that it will meet the critical expectations of professional experts. A number of articles have been added to the pharmacopeia, and almost an equal number have been dismissed from its pages. A considerable list of changes of official Latin titles is announced, and about treble the number of English titles have been changed. The metric system has been adhered to with reference to weights and measures throughout the volume. This action of the committee will do much to hasten the adoption of the metric system by physicians and druggists, though it will occasion some perplexity among those unfamiliar with its uses and equivalents. A table of equivalents of weights and measures—customary and metric—is given on page 554, *et. seq.*, for reference by those who may need it.

The general make-up of the volume is such as to commend it to those who may have occasion to consult it. The titles are in large, heavy type and the descriptions are printed in heavy small pica, while further allusion to each article appears in strong-faced long primer type. The book has been somewhat delayed in its appearance, but this could hardly be avoided when the extraordinary labors of the committee are taken into consideration. The changes are more radical in this revision than in any of its predecessors, consequently greater care has been necessary in its publication, and hence, more time was required to do the work satisfactorily.

---

TRANSACTIONS OF THE AMERICAN SURGICAL ASSOCIATION. Volume XI. Edited by DEFOREST WILLARD, M. D., Recorder of the Association. Philadelphia: Printed for the Association, and for sale by William J. Dornan. 1893.

This volume records the work of this celebrated association done at its meeting in Buffalo last May. While it is not as large as some of its predecessors, it is yet replete with articles of great interest. The president, Dr. Nicholas Senn, chose for the subject of his address, A New Method of Direct Fixation of the Fragments in Compound and Ununited Fractures, in which he discusses the history of direct immobilization of fragments, and brings to the notice of the profession his own plan of the retention of compound ununited fractures by direct fixation with bone ferrule. This consists of preparing from the bones of animals a ferrule, varying from a quarter of an inch to an inch in width, and about one-sixth of an inch in thickness, or, in some instances, a much thinner ring may furnish the necessary lateral support. Sterilization is effected by boiling for an hour or more, after which the rings are kept immersed in sublimate alcohol 1–1000. Under strict antiseptic precautions the seal of fracture is to be exposed in such a way that both fragments are readily accessible. The most convenient fragment is isolated, the ferrule slipped over it and pushed away from the line of fracture far enough to clear the other fragment. After reduction is accomplished, the second fragment is engaged in the ring, which is then pushed back sufficiently far to grasp both fragments securely. Bending at the seat of fracture is prevented by the application of a plaster of Paris splint, fenestrated at a point opposite the wound. Dr. Senn reported a number of cases in which he had employed this method with success. This unique address is illustrated with twenty-three wood-cuts, and is one of the most valuable contributions to the literature of surgery of the year. The volume is full of interest, and should be in the hands of every practical surgeon.

---

A Practical Treatise on the Diseases of the Skin. For the use of students and practitioners. By J. Nevins Hyde, A. M., M. D., Professor of Dermatology and Venereal Diseases in Rush Medical College, Chicago. New (third) edition. In one octavo volume of 802 pages, with nine plates, of which three are colored, and 108 engravings. Cloth, $5.00; leather, $6.00. Philadelphia: Lea Bros. & Co. 1893.

The science of dermatology has advanced so rapidly that a work written ten years ago, like the present one under consideration, even with a new edition five years thereafter, finds it necessary after five years more to rewrite a large part of the original treatise as well as to add many new and important chapters. Dr. Hyde is an experienced teacher as well as a competent author, and his former editions were received with approval by dermatologists as well as by those general practitioners who are interested in the study and treatment of diseases of the skin.

To illustrate, Hyde states in his preface that thirty-five new diseases are considered in the present edition; that the chapter on tuberculosis has been entirely rewritten and enlarged to meet the most modern views of this subject, and that several other important changes have been made for like reasons. There are two chapters that will prove of especial interest to dermatologists and general practitioners alike, namely—one on general therapeutics, in which the nature and application of various drugs used in treatment of diseases of the skin are described, and another entitled Dermatitis Medicamentosa, in which a large number of drugs are catalogued that cause cutaneous eruptions with greater or less frequency. This list is constantly being added to, as experience demonstrates the tendency among drugs to cause eruptions, and it is probable that in another edition Hyde will be called upon to considerably increase the category, as he himself intimates.

A large number of new and original illustrations—five plates and twenty-two wood-cuts—have been designed especially for this edition. The mechanical execution of the work is all that could be desired, and the treatise is one that affords much satisfaction, in that it is a safe guide for both students and practitioners, either general or special, and particularly does it adapt itself to the use of dermatologists.

---

THE PRINCIPLES AND PRACTICE OF SURGERY. By JOHN ASHHURST, Jr., M. D., Barton Professor of Surgery and Clinical Surgery in the University of Pennsylvania; Surgeon to the Pennsylvania Hospital, Philadelphia. New (sixth) edition, enlarged and thoroughly revised. In one octavo volume of 1,161 pages, with 656 engravings and a colored plate. Cloth, $6.00; leather, $7.00. Philadelphia: Lea Bros. & Co. 1893.

This author has been before the surgical world so long and is so versatile and resourceful, that his several editions are rapidly taken up and others follow in equally prompt measures of time. Ashhurst has taken great pains to render this sixth edition fully equal to the demands of the present, and has constructed it on lines which merit a continuance of the confidence of the profession. In this edition he has incorporated an account of the more important recent observations in surgical science, as well as such novelties in surgical practice as merit the classification of improvements. Dr. Charles B. Nancrede, of Ann Arbor, has contributed a new chapter on surgical bacteriology; Dr. Barton C. Hirst has revised the sections on gynecological subjects; and Drs. George

E. de Schweinitz and Alexander Randall have revised the chapters on diseases of the eye and ear. The volume preserves the same general arrangement as in former editions, but by the exclusion of matter that has ceased to be of importance, much space has been gained, permitting the introduction of a large amount of new material, with only a slight increase in the total number of pages.

The illustrations, too, have been greatly improved by the introduction of original cuts from photographs, and a colored plate containing seven figures pertaining to bacteriological subjects. We think it important in the present age that surgical treatises should be more completely illustrated by photographs from actual cases than is generally practised. Some of the cuts in this book are old-timers, that might well be replaced by a further use of the photograph.

We desire to especially compliment the author on his index, which increases the value of the work for reference by its completeness. There is nothing new or of special interest in the gynecological part of the work, and we think that it would be quite as well for surgeons to omit this department in their treatises and so be enabled to devote more space to the consideration of surgical conditions that are often too condensed to be of great value to surgeons or students of surgery.

Those surgeons who possess earlier editions of Ashhurst's Treatise will make haste to obtain this new one, and those who are not familiar with the work will necessarily add it to their libraries. The surgical science is so varied and extensive in its application, that one must needs have at hand all the contemporary authors extant in order to intelligently keep pace with its progress.

---

A TEXT-BOOK OF PHYSIOLOGICAL CHEMISTRY. By OLOF HAMMARSTEN, Professor of Medical and Physiological Chemistry in the University of Upsala. Authorized translation from the second Swedish edition and from the author's enlarged and revised German edition. By John A. Mandel, Assistant to the Chair of Chemistry, etc., in the Bellevue Hospital Medical College and in the College of the City of New York. First edition; first thousand. Octavo, cloth, pp. x.—511. Price, $4.00. New York: John Wiley & Sons, 53 East Tenth street. 1893.

The medical profession is to be congratulated upon having received another addition to the meager literature on physiological chemistry. It indicates an advance and a demand for a more thorough study of chemistry in its relations to physiological processes.

This volume, from the pen of a man of international reputation, gives an excellent exposition of the subject of physiological chemistry. We believe, however, that the book would have been very much enhanced in value if the subject of pathological chemistry had been treated in more thorough detail. It is to be regretted that the author did not see fit to allow Mr. Mandel to make such additions as would bring this branch of the subject up to date. While the physician should be familiar with the normal conditions from a chemical standpoint, it is also of vital importance that he should be familiar with any morbid declensions from the normal performance of the functions of any of the organs of the animal economy.

The typography of the book is excellent, it is very neatly bound, and quite free from errors. It is a decided credit to the publishers. J. A. M.

---

The National Dispensatory. Containing the Natural History, Chemistry, Pharmacy, Actions and Uses of Medicines, including those recognized in the Pharmacopeias of the United States, Great Britain and Germany, with numerous references to the French Codex. By Alfred Stille, M. D., LL. D., Professor Emeritus of the Theory and Practice of Medicine and of Clinical Medicine in the University of Pennsylvania; John M. Maisch, Ph. M., Phar. D., Late Professor of Materia Medica and Botany in Philadelphia College of Pharmacy; Secretary to the American Pharmaceutical Association; Charles Caspari, Jr., Ph. G., Professor of Pharmacy in the Marland College of Pharmacy, Baltimore; and Henry C. C. Maisch, Ph. G., Ph. D. New (fifth) edition, thoroughly revised, according to the new United States Pharmacopeia (seventh decennial revision, 1894). In one magnificent imperial octavo volume of 1910 pages, with 320 elaborate engravings. Cloth, $7.15; leather, $8.00. With ready reference thumb-letter index—cloth, $7.85; leather, $8.50. Philadelphia: Lea Brothers & Co. 1894.

That this great work should present itself to the profession within five months after the publication of the new United States Pharmacopeia, and but a single month after it went into effect, must afford great satisfaction to the editors and publishers. But it is also of equal satisfaction to the professions of medicine and pharmacy, whose interests are so intimately blended in the National Dispensatory. The vast fund of information which it contains should be placed at the disposal of all concerned with the least possible delay. It is the official guide for the medical and pharmaceutical professions, and is to them of like importance as the rudder to the great ocean steamship in buffeting the storms and high seas of practical medicine and pharmacy.

The changes in the new Pharmacopeia, to which we have alluded elsewhere, were of such a sweeping character that it necessarily entailed greater work upon the part of authors and editors to prepare this edition than upon those that had preceded it. New tables of value have been inserted, weights and measures are given with the ordinary and metric systems, and adequate space has been allotted to the new synthetic compounds, as well as to the unofficial preparations that are now so extensively used. The volume, too, is rich in chemical and pharmaceutical information, with data, formulas and tables gathered from all official sources. The latest editions of foreign pharmacopeias have been summoned to pay tribute to this wonderful work. Its descriptions of the materia medica are clear, thorough and systematic, and these characteristics apply equally to its explanations of chemical and pharmaceutical processes and tests.

We must not fail to make mention of the therapeutical portion of the work, which challenges admiration for its completeness, while the statements of the actions and uses of medicine, arranged alphabetically in the text under the names of the drugs, are placed most easily and suggestively at command by the recommendations under the various diseases in the therapeutical index. There has been also a free use of illustrations wherever they can be made valuable in aiding the description of drugs, or of the most approved apparatus. The two indexes, general and therapeutical, cover twenty-five thousand references, and the total number of pages exceed nineteen hundred. A thumb index is added for the convenience of those who choose to pay the slight additional charge of fifty cents.

This work will find its way into every pharmacy of the land and to the book shelves of teachers of materia medica; and a large army of general practitioners cannot afford to do without it. Orders should be placed promptly, as the demands for the book will be very great.

---

Transactions of the Medical Association of Georgia. Forty-fourth Annual Session, 1893. Octavo, pp. 426. Atlanta, Ga.: Published by the Association. 1893.

This book furnishes ample evidence of the substantial progress making by the medical profession in the Empire State of the South. The papers contained in this volume are for the most part of exceptional quality, and some of the discussions are of an exceedingly interesting character. The contagiousness of consumption, by Dr. J. G. Hopkins, Thomasville, is a paper of great practical interest, and emphasizes the importance of strict attention to preventive measures. A paper entitled Impure and Pure Mineral Waters, by Dr. T. S. Hopkins, of Thomasville, also contains many points of interest. The Technique and After Treatment of Ovariotomy, by J. B. S. Holmes, of Rome, deserves to be carefully studied by every abdominal surgeon. It is classified under sub-heads, and together with the discussion covers thirty-four pages of the book. There are also interesting papers by prominent authors, such as Gaston, Westmoreland, McRae and others that we cannot give the space they deserve in this brief notice. The book is printed on heavy paper, handsomely bound, and will easily find its way into the hands of every progressive physician in Georgia, as well as many outside that commonwealth.

---

SANDERS' QUESTION COMPENDS, NO. 12. Essentials of Minor Surgery, Bandaging and Venereal Diseases, arranged in the form of questions and answers, prepared especially for students of medicine. By EDWARD MARTIN, A. M., M. D., Clinical Professor of Genito-Urinary Diseases; Instructor in Operative Surgery and Lecturer on Minor Surgery, University of Pennsylvania; Surgeon to the Howard Hospital; Assistant Surgeon to the University Hospital, etc., etc., Second edition, revised and enlarged, seventy-eight illustrations. Philadelphia: W. B. Sanders. 1893.

The first edition of this number of the question compends was so well received that it soon became exhausted. This second edition, according to the author's preface, has been thoroughly revised and brought up to the present standard of surgical practice. A considerable number of the illustrations have been redrawn and engraved, and an entirely new set of bandaging cuts inserted. These latter are to be especially commended, and Dr. Martin acknowledges his indebtedness to the *American Text-Book of Surgery* for them as well as for the descriptions given. If there is an excuse for this kind of literature, it is readily to be found in such a good work as this.

---

A MANUAL FOR BOARDS OF HEALTH AND HEALTH OFFICERS. By LEWIS BALCH, M. D., Ph. D., Secretary State Board of Health of New York; Health Officer of Albany; Emeritus Professor of Anatomy and Professor of Medical Jurisprudence Albany Medical College. Pp. 242. Albany, N. Y.: Banks & Bros. 1893.

The author of this manual has had a large experience in reference to the duties of health officers and of boards of health, therefore, is unusually well equipped for the preparation of such a book. He announces that his object has been to put into the hands of health boards and health officers a short compact guide to follow in the discharge of their duties. It is not written from a legal standpoint, nor is it intended to instruct in hygiene, but is simply a practical statement of the duties of health authorities and how they may be performed under the public health law of the State of New York. It was prepared to supply a demand on the part of those engaged in the public health service, and admirably fulfils the purposes for which it is published.

We notice that instruction in detail is given with reference to tuberculosis in cattle, with full directions regarding the care and destruction of animals so diseased, but we think that an additional precaution should be taken by compelling the cremation of all tuberculous carcasses.

---

MINERAL SPRINGS AND HEALTH RESORTS OF CALIFORNIA, with a complete Chemical Analysis of every Important Mineral Water in the World. Illustrated. A Prize Essay. Annual Prize of the Medical Society of the State of California, awarded April 20, 1889. By WINSLOW ANDERSON, M. D., M. R. C. P., Lond., M. R. C. S., Eng., etc.; Joint Editor and Publisher of the *Pacific Medical Journal*, etc., etc. San Francisco: The Bancroft Co. 1892.

An examination of this book impresses the reader with the fact that California is wonderfully equipped in the number and quality of its mineral springs. To collect information on the subject and put it into book-form must have been an interesting occupation for the author of this book. Within its pages information can be obtained as to the relative value, medicinally speaking, of the several springs in the golden state. These have been carefully analyzed for the most part by the author, and tables of the analysis are published. In the majority of instances, health resorts have been erected contiguous to the springs, and illustrations thereof are published in the book. Those who desire information on the subject will readily find it by consulting this volume.

---

BUREAU OF EDUCATION, (Whole number, 195) CIRCULAR OF INFORMATION, NO. IV., 1893. Abnormal Man: being Essays on Education and Crime and Related Subjects, with Digests of Literature and a Bibliography. By ARTHUR MACDONALD, Specialist in the Bureau of Education. Washington: Government Printing Office. 1893.

This volume is made up of two sections. The first, consisting of 204 pages, divided into eight chapters. Chapter I. considers education in its relation to crime, including comparative statistics of crime and education in France, Italy, Germany, Austria, Japan and the United States. Chapter II. deals with practical criminology, including discipline and instruction. Chapter III. treats of the Mafia in a most instructive way. Chapter IV. discusses alcoholism and crime. Chapter V. is very instructive and entertaining, dealing, as it does, in the foibles and eccentricities of the great geniuses. Chapter VI. is sociological and ethical in character. The remainder of the book is composed of 200 pages of carefully compiled bibliography. Altogether, the Bureau presents us a most valuable *résumé* of the subject to date, and Mr. McDonald is to be congratulated on the excellence of the work and its admirable arrangement. J. W. P.

### BOOKS RECEIVED.

Venereal Memoranda. A Manual for the Student and Practitioner. By P. A. Morrow, A. M., M. D. Clinical Professor of Venereal Diseases in the University of the City of New York; Surgeon to Charity Hospital; Attending Surgeon to the Bellevue Hospital Out-Door Relief, Department of Skin Diseases; Member of the American Dermatological Association; Member of the New York Dermatological Society, etc., etc. Double duodecimal, pp. iv.—332. New York: William Wood & Co. 1894.

The Johns Hopkins Hospital Reports. Report in Gynecology, 11. Volume III.; Nos. 7, 8, 9. Imperial octavo, pp. 461. Baltimore: The Johns Hopkins Press. 1894.

The Physician's Wife, and the Things that Pertain to Her Life. By Ellen M. Firebaugh. With portrait of author and 44 photo-engravings of original sketches. In one crown octavo volume of 200 pages. Extra cloth, $1.25 net. Special limited edition, first 500 copies, numbered, and printed in photogravure ink, on extra fine enameled paper; bound in half-leather and Vellum cloth, $3.00 net. Philadelphia: The F. A. Davis Co., Publishers, 1914 and 1916 Cherry street.

The Modern Climatic Treatment of Invalids with Pulmonary Consumption in Southern California. By P. C. Remondino, M. D. Member of the American Medical Association, American Public Health Association, etc., etc. Physicians' Leisure Library. Detroit, Mich.: George S. Davis. 1893. Price, paper, 25 cents; cloth, 50 cents.

A Practical Treatise on the Diseases of the Hair and Scalp. By George Thomas Jackson, M. D. Professor of Dermatology, Woman's Medical College, New York Infirmary; Chief of Clinic and Instructor in Dermatology, College of Physicians and Surgeons; Consulting Dermatologist, Presbyterian Hospital; Visiting Dermatologist, Randall's Island Hospital; Member of the American Dermatological Association, etc. New revised and enlarged edition. Small 8vo, pp. 414. New York: E. B. Treat, 5 Cooper Union. 1893. Price, $2.75.

A Treatise on Headache and Neuralgia, including Spinal Irritation and a Disquisition on Normal and Morbid Sleep. By J. Leonard Corning, M. A., M. D. Consultant in Nervous Diseases to St. Francis' Hospital; Fellow of the New York Academy of Medicine; Member of the

New York Neurological Society, etc. With an Appendix. Eye Strain, Cause of Headache. By David Webster, M. D., Professor of Ophthalmology in the New York Polyclinic; Surgeon to the Manhattan Eye and Ear Hospital, etc., etc. Illustrated. Third edition. Small 8vo, pp. 275. New York: E. B. Treat, 5 Cooper Union. London: H. K. Lewis, 136 Grower street. 1894. Price, $2.75.

Treatment of the Diseases of the Stomach and Intestines. By A. Mathieu, Physician to the Paris Hospitals. (Medical Practitioners' Library.) Octavo, 285 pages. Parchment muslin, price, $2.50; flexible leather, gilt top, price, $3.25. New York: William Wood and Company. 1894.

Operative Surgery. By Th. Kocher, M. D., Professor at the University and Director of the Surgical Clinic at the Berne University. Octavo, 288 pages, 163 illustrations. Extra muslin, price, $3.00. New York: William Wood and Company. 1894.

Proceedings of the Philadelphia County Medical Society. Volume XIV. Session of 1893. Lewis H. Adler, Jr., M. D., Editor. Octavo, pp. xxviii.—484. Philadelphia: William J. Dornan. 1893.

Holden's Anatomy. A Manual of the Dissections of the Human Body. By John Langton, F. R. C. S., Surgeon to, and Lecturer on, Anatomy at St. Bartholomew's Hospital. Carefully revised by A. Hewson, M. D., Demonstrator of Anatomy, Jefferson Medical College; Chief of Surgical Clinic, Jefferson Hospital; Member Association American Anatomists, etc. 311 illustrations. Small 8vo, pp. xx.—803. Philadelphia: P. Blakiston, Son & Co., 1012 Walnut street. 1894. Price, $3.00.

Antiseptic Therapeutics. By Dr. E. L. Trouessant, Paris, France. Translated by E. P. Hurd, M. D. In two volumes. Physicians' Leisure Library. Detroit, Mich.: George S. Davis. 1893. Price, paper, 25 cents each; cloth, 50 cents each.

A Text-Book of the Theory and Practice of Medicine. By American teachers. Edited by William Pepper, M. D., LL. D., Provost and Professor of the Theory and Practice of Medicine and of Clinical Medicine in the University of Pennsylvania. In two volumes; illustrated. Vol. II. Large octavo, pp. xii.—1046. Philadelphia: W. B. Saunders, 913 Walnut street. 1894. Price per volume, cloth, $5.00; leather, $6.00; half Russia, $7.00. For sale by subscription only.

An Illustrated Encyclopedic Medical Dictionary. Being a dictionary of technical terms used by writers on medicine and the collateral sciences, in the Latin, English, French, and German languages. By Frank P. Foster, M. D., editor of the *New York Medical Journal*, assisted by eleven collaborators. Vol. IV. Minn-Zyth. With illustrations. Quarto, pp. 776. D. Appleton & Co. 1894.

Lectures on Auto-Intoxication in Disease, or Self-Poisoning of the Individual. By Ch. Bouchard, Professor of Pathology and Therapeutics, Member of the Academy of Medicine, and Physician to the Hospitals, Paris. Translated, with a preface, by Thomas Oliver, M. A., M. D., F. R. C. P., Professor of Physiology, University of Durham; Physician to the Royal Infirmary, Newcastle-upon-Tyne; and Examiner in Physiology, Conjoint Board of England. In one octavo volume; 302 pages. Extra cloth, $1.75 net. Philadelphia: The F. A. Davis Co., Publishers, 1914 and 1916 Cherry street.

